# Matrix Metalloproteinase‐Responsive Controlled Release of Self‐Assembly Nanoparticles Accelerates Heart Valve Regeneration In Situ by Orchestrating Immunomodulation

**DOI:** 10.1002/advs.202403351

**Published:** 2024-11-13

**Authors:** Xing Chen, Yunlong Wu, Peng Song, Liandong Feng, Ying Zhou, Jiawei Shi, Nianguo Dong, Weihua Qiao

**Affiliations:** ^1^ Department of Cardiovascular Surgery Union Hospital Tongji Medical College Huazhong University of Science and Technology Wuhan 430022 China; ^2^ Department of Cardiovascular Surgery Zhongnan Hospital Wuhan University Wuhan Hubei 430071 China; ^3^ School of Chemistry and Engineering Huazhong University of Science and Technology Wuhan Hubei 430074 China; ^4^ Hubei Provincial Key Laboratory of Occurrence and Intervention of Rheumatic Diseases Minda Hospital of Hubei Minzu University Enshi 445000 China

**Keywords:** hydrogel, macrophage polarization, microRNA, nanoparticles, tissue engineering heart valves

## Abstract

In situ tissue engineering heart valves (TEHVs) are the most promising way to overcome the defects of existing valve prostheses. Despite their promising prospects, the clinical translation of TEHVs remains a formidable challenge, mainly due to unpredictable host interactions post‐implantation. An immunomodulatory idea based on hydrogel encapsulation of nanoparticle‐coated heart valve scaffolds is introduced. Specifically, galactose‐modified human serum albumin nanoparticles (miR‐93@HSA NPs) to deliver microRNA‐93 mimics are utilized, which target macrophages and induce their differentiation into the anti‐inflammatory M2 subtype, fostering a conducive immune microenvironment. Matrix metalloproteinase (MMP)‐responsive hydrogel is used to encapsulate the nanoparticles, enabling targeted and sustained release. Results show that the miR‐93@HSA NPs exhibit excellent ability to induce macrophage polarization toward the M2 phenotype. A decellularized valve modified with hydrogel reveals MMP‐response release of the miR‐93@HSA NPs. In vitro, the immunomodulatory heart valve possesses good endocytocompatibility and effectively reprograms macrophages when cocultured with HUVECs or RAW264.7 macrophages. In vivo, this valve scaffold promises to mitigate early inflammatory damage and provide a pro‐endothelialization niche for scaffolds' constructive remodeling. With the use of cell coculture systems and transcriptome sequencing, the mechanism of immune‐modulating scaffold accelerating endothelialization is being elucidated. The immunomodulatory heart valve scaffold holds promising potential for clinical translation.

## Graphical abstract

After implantation of the immunoregulatory scaffold into the body, infiltrated immune cells produce MMP, enabling the degradation of MMP‐responsive hydrogels. Subsequently, galactose‐modified nanoparticles are exposed and phagocytosed by macrophages, triggering their transformation into an anti‐inflammatory phenotype. This process offers the potential to alleviate early inflammatory damage and foster an immune microenvironment favorable for the early re‐endothelialization of scaffolds.

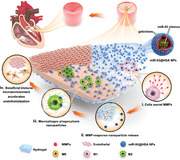

## 1 Introduction

Valvular heart disease (VHD) has a high morbidity and mortality risk, posing a serious threat to human health.^[^ ^1^ ^]^ Surgical intervention, particularly prosthetic valve replacement, remains the most effective treatment for VHD. Current clinical application of valve replacement is still mainly mechanical and bioprosthetic valve, both of which have notable limitations. TEHVs offer a more promising alternative, overcoming the limitation of commercial artificial valves, with the ability to grow with patients and repair itself.^[^ ^2^, ^3^ ^]^ In situ regeneration, which involves the direct implantation of a bare scaffold into the body to facilitate recellularization, self‐repair, and eventual regeneration into a native valve,^[^ ^4^ ^]^ offers a more feasible approach for clinical translation.

Decellularized valve scaffold (DVS), obtained from either homogenic or xenogeneic natural heart valve undergoing a meticulous decellularization process, retains its native morphology and original three‐dimensional structure while minimizing immunogenicity, thereby providing cues to achieve tissue regeneration.^[^ ^5^ ^]^ However, in the absence of endothelial protection, collagen becomes fully exposed to the blood environment, in turn leading to thrombogenesis and chronic inflammatory response.^[^ ^6^ ^]^ Given the DVS's immersion in a dynamic blood flow environment, thrombosis poses an inherent challenge. Fortunately, hydrogel modification effectively alters the rough surface morphology of DVS, significantly reducing plasma protein adsorption, and delaying platelet adhesion and activation, ultimately mitigating the threat of thrombosis.^[^ ^7^ ^]^

The immune response, particularly involving macrophages, holds a pivotal role in scaffold regeneration, yet its profound implications for heart valve regeneration remains unexplored. The infiltration of immune cells augments material remodeling, whereas cytokine secretion triggers migration, proliferation, and differentiation of adjacent endothelial cells or stem cells, ultimately fostering scaffold endothelialization. Macrophages, whose functionality is contingent on specific subtypes, persist throughout the immune response subsequent to biomaterial implantation.^[^ ^8^ ^]^ Depending on their activation status, macrophages can be categorized into two types: the pro‐inflammatory M1 subtype and the anti‐inflammatory M2 subtype. Mountains of studies have shown that inhibiting M1 and promoting M2 differentiation can mitigate organ damage caused by inflammatory response and promote tissue repair.^[^ ^9^, ^10^ ^]^ In early inflammation period, monocytes pioneer the entry into scaffolds, transforming into M1 macrophages that unleash a cascade of pro‐inflammatory factors like TNF‐α and IL‐6, accelerating cellular migration and secreting VEGF to promote scaffold cellularization. During the later stage of proliferation, M2 macrophages release anti‐inflammatory factors and growth factors such as IL‐10, TGF‐β, and IGF‐1, thereby promoting scaffolds remodeling. Therefore, how to utilize the balance between macrophage subtypes to reconcile the contradiction between remodeling and inflammation is the key to achieving in‐situ regeneration of the scaffolds.^[^ ^11^ ^]^

In this study, we delved into the temporality of immune regulation, meticulously examining the evolving immune microenvironment in both subcutaneous implantation and abdominal aortic transplantation. Accordingly, we innovatively introduce an MMP‐response hydrogel system in which infiltrating immune cells produce the MMP to degrade hydrogel and engulf nanoparticles to convert to anti‐inflammatory phenotype; This system strategically accounts for varying M1/M2 macrophage ratios, enabling a time‐sequential manner.^[^ ^12^, ^13^ ^]^ Galactose‐modified nanoparticles were used to specifically target macrophage, creating an immune‐modulating scaffold. By incorporating miR‐93‐loaded nanoparticles and MMP‐responsive hydrogel into the DVS modification, this scaffold holds the promise of mitigating early inflammatory damage, providing an immune microenvironment conducive to the early re‐endothelialization of scaffolds, and ultimately promoting the constructive remodeling of TEHV eventually.

## 2 Results

### 2.1 Preparation and Characterization of miR‐93‐PEG‐HSA NPs

**Figure 1:**
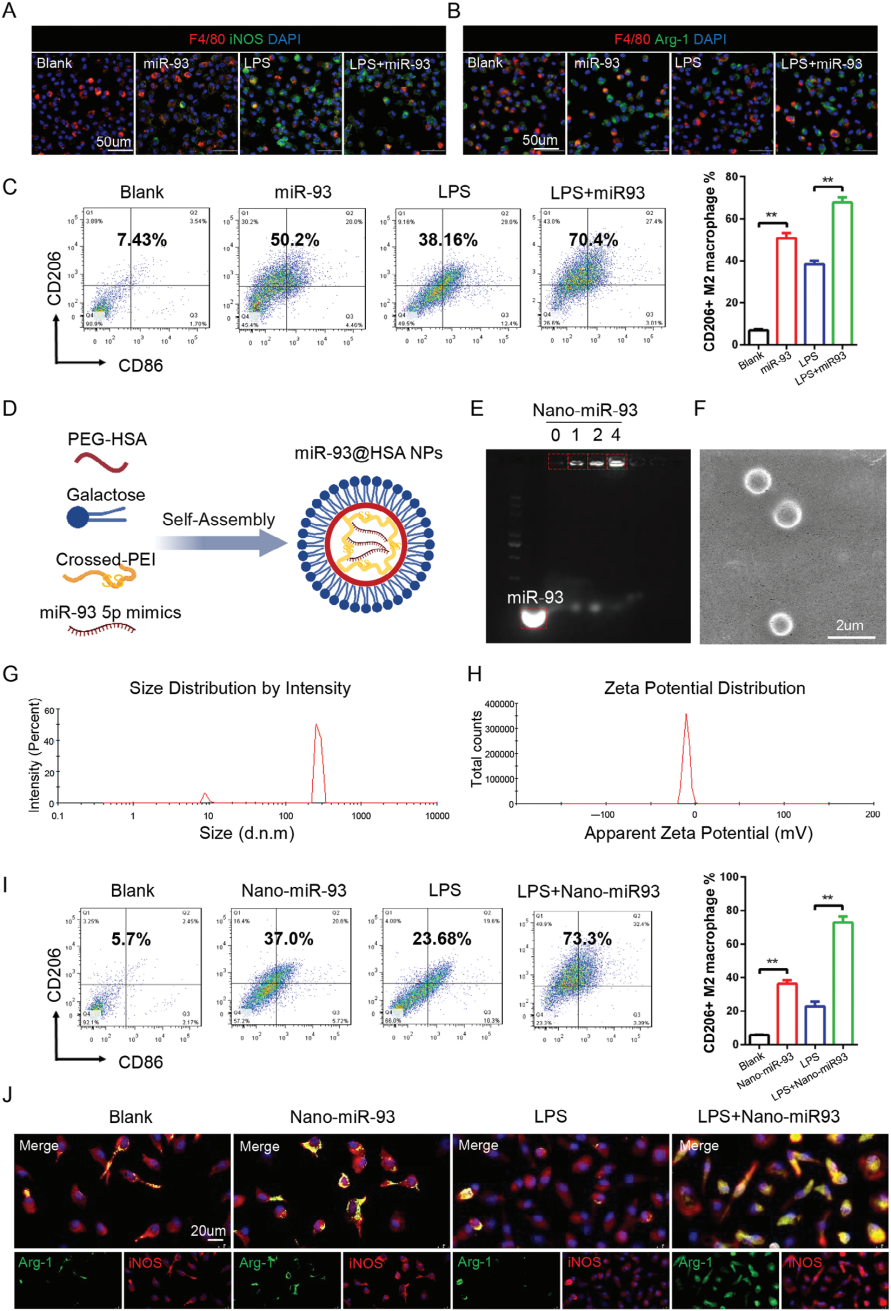
Preparation and Characterization of miR‐93‐PEG‐HSA NPs. A,B) Immunofluorescence staining of the BMDMs. A), (CD68; red), (iNOS; green); B), (CD68; red), (Arg‐1; green). Scale bars = 50 µm. C) Representative graphs of CD206+ M2 macrophages. n = 3. D) Schematic illustration of miR‐93 5p mimics loaded PEG‐HSA nanoparticles prepared using self‐assembly method. E) Representative image of agarose gel electrophoresis of free or wrapped miR‐93 5p mimics. F) Representative SEM image of miR‐93@HSA NPs. Scale bars = 2 µm G) Size distribution of nanoparticles. H) Zeta potential distribution of nanoparticles. I) Representative graphs of CD206+ M2 macrophages. Scale bars = 20 µm. *n* = 3. J) Immunofluorescence staining of the BMDMs. (iNOS; red), (Arg‐1; green). Data were expressed as mean ± SD, ^*^ *p *< 0.05, ^**^ *p *< 0.01, ^***^ *p *< 0.001.

Previous studies conducted microRNAs such as miR‐21, miR‐93, miR‐181, and miR‐223 could promote macrophage polarizing toward M2.^[^ ^14^ ^]^ Among these, miR‐93 was selected for nanoparticle formulation due to its pronounced ability to augment M2 polarization in macrophages, while exerting minimal influence on endothelial cell proliferation (Figure S1, Supporting Information). In this study, the effects of miR‐93 on the macrophage polarization were assessed in vitro. Immunofluorescence staining showed that BMDMs treated with miR‐93 5p mimics decreased the expression of iNOS and increased the Arg‐1 level (**Figure** **1**A,B). Flow cytometry also exhibited miR‐93 treatment significantly elevated the ratio of M2 cells with or without LPS and IFN‐γ intervention (Figure 1C).

In order to improve the delivery efficiency of miR‐93, we devised a robust, macrophage‐specific, self‐assembled nanoparticle delivery system. This innovation led to a significant improvement in the efficacy of miR‐93 delivery. Currently, galactose‐modified and folate‐modified targeted macrophage nanoparticles are widely used in research.^[^ ^15^, ^16^ ^]^ Since folic acid has a strong yellow color, and the efficiency of nanoparticles phagocytosed by macrophage and drug encapsulation rate was detected according to the fluorescence intensity of FAM carried by miR‐93 5p mimics, the use of folic acid modification would bring great difficulties in detection. Therefore, galactose‐modified nanoparticles were used in this study. The negatively charged miR‐93 is mixed with positively charged crosslinked poly(ethylene imine) (cPEI) to form particles through ionic interactions. Separately, when galactose is mixed with PEG‐modified HSA, the hydrophobic moieties of both molecules bind together, effectively modifying the galactose onto the surface PEG‐HSA. Then the preparation of miR‐93‐cPEI‐Galactose‐PEG‐HSA NPs (miR‐93@HSA NPs) was fabricated utilizing a layer‐by‐layer self‐assembly method (Figure 1D). Agarose‐gel electrophoresis revealed that the free miR‐93 migrated toward the positive electrode, whereas the miR‐93 loaded within the nanoparticles remained in proximity to the negative terminal (Figure 1E). In order to improve drug loading efficiency, we gradually increased the ratio of miR‐93 to cPEI, and found that the optimal miR‐93/cPEI was 4:1 (Figure 1E). miR‐93@HSA NPs exhibited a compact spherical morphology with scanning electron microscopy (SEM) (Figure 1F). Dynamic light scattering indicated that the size of the miR‐93@HSA NPs is mainly distributed in 200–400 nm, and the charge is mainly distributed in ‐2 ∼ 0 mV (Figure 1G and H).

Flow cytometry was used to detect the phagocytosis efficiency of miR‐93‐FAM@HSA NPs. Macrophages have phagocytic ability, and the phagocytic rate of the miR‐93‐FAM@HSA NPs with 0%, 5% 10%, and 20% galactose modification is 22.6 ± 0.608%, 44.8 ± 2.11%, 53.27 ± 0.933%, and 57.3 ± 1.429%, respectively. Although the phagocytic efficiency of 20% galactose modification was slightly higher than that of 10% galactose modification, in order to take into accounting the drug loading efficiency, the concentration of 10% galactose modification was finally selected in this study (Figure S2A, Supporting Information). In addition, compared with HUVECs and VSMCs, macrophages showed an obvious phagocytic efficiency advantage for 10% galactose‐modified nanoparticles (Figure S2B, Supporting Information). The effect of miR‐93@HSA NPs on macrophage polarization was also evaluated. Immunofluorescence and flow cytometry showed excellent ability to induce BMDMs M2 polarization of miR‐93@HSA NPs (Figure 1I,J).

### 2.2 Construction of the Immune‐Modulating Scaffold

**Figure 2:**
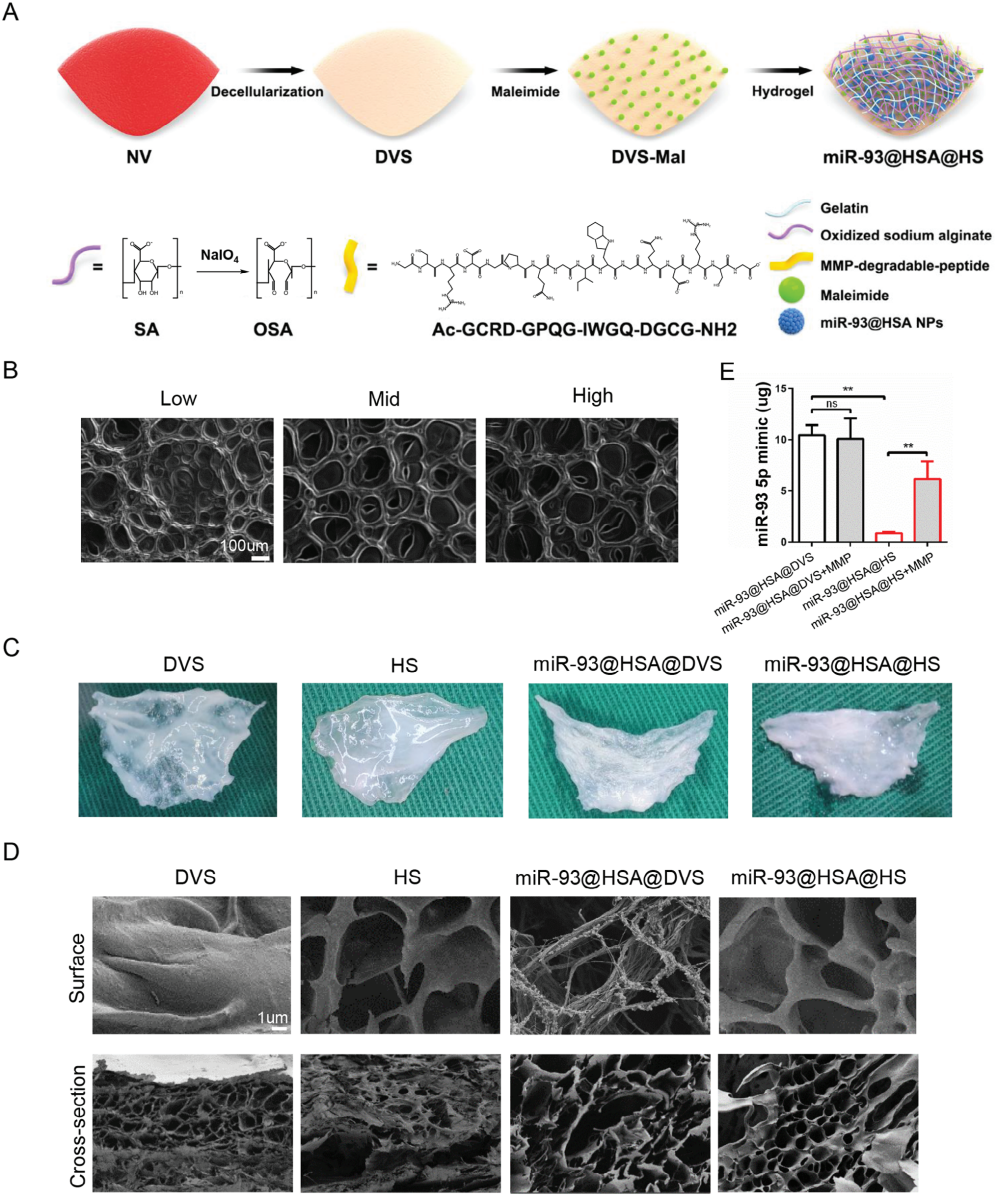
Construction of the immune‐modulating scaffold. A) Schematic picture of preparing the immune‐modulating scaffolds. B) SEM shows the representative picture of the hydrogels of different oxidation degree. Scale bars = 100 µm. C) General view of the different scaffolds. D) SEM showing the topology of the surface and the cross‐section in different scaffolds. Scale bars = 1 µm. E) MMP‐response release of nanoparticles encapsulated in scaffolds detected by a multimode microplate reader. *n* = 3. Data were expressed as mean ± SD, ^*^ *p *< 0.05, ^**^ *p *< 0.01, ^***^ *p *< 0.001. DVS: decellularized valve scaffolds; miR‐93@HSA@DVS: miR‐93@HSA nanoparticles coated DVS; HS: hydrogel coated DVS; miR‐93@HSA@HS: hydrogel and miR‐93@HSA nanoparticles coated DVS.

In order to achieve the macrophage‐targeted release and slow release of miR‐93@HSA NPs, a double cross‐linked hydrogel of oxidized sodium alginate (OSA) ‐amino gelatin was constructed in this study. OSA was grafted with matrix metalloproteinase (MMP) substrate peptide, which could achieve MMP‐responsive degradation of OSA‐_MMP_/AG hydrogel.^[^ ^17^ ^]^ The other end of the grafted substrate peptide was connected with the maleimide‐modified DVS through the sulfhydryl to make encapsulation of the DVS modified with miR‐93@HSA NPs (**Figure** **2**A). The oxidation degree of oxidized sodium alginate largely determines its porosity. SEM showed that the hydrogel prepared with high oxidation degree sodium alginate (the high group) had larger pores, and it was used in the subsequent hydrogel preparation (Figure 2B). The general view of scaffolds from different group is shown in Figure 2C. Scaffolds from porcine natural aortic valves showed a white lobular structure (DVS group), after modified with hydrogel presented a thin layer of transparent gel (HS group and miR‐93@HSA@HS group). Micromorphological analysis revealed that, following hydrogel modification, uneven fibrous structure of DVS changed to a mesh structure, and the scaffold modified with miR‐93@HSA NPs surface can be seen with a diameter of 200–300 nm microsphere structure (Figure 2D). Hydrogel coating or NPs modification has no obvious effects on the cross‐sections appearance of scaffolds, which maintained their loose and porous honeycomb structures.

In order to explore the MMP response of the scaffolds, this study used MMP2 protein to intervene in the valve composite, and then detected the release of nanoparticles (Figure 2E). The results showed that when MMP2 was not added, compared with miR‐93@HSA@DVS group (NPs contained 10.44 ± 0.585 µg of miR‐93), miR‐93@HSA@HS group (NPs contained 0.8948 ± 0.063 µg of miR‐93) had a lower nanoparticle release rate. When MMP2 was added, the valve material of miR‐93@HSA@HS group (the miR‐93 contained in NPs was 6.167 ± 1.003 µg) showed significantly increased release of nanoparticles at 24, while that of miR‐93@HSA@DVS group (the miR‐93 contained in NPs was 10.10 ± 1.163 µg) showed no significant change.

### 2.3 In Vitro Hemocompatibility of the Immune‐Modulating Scaffold

**Figure 3:**
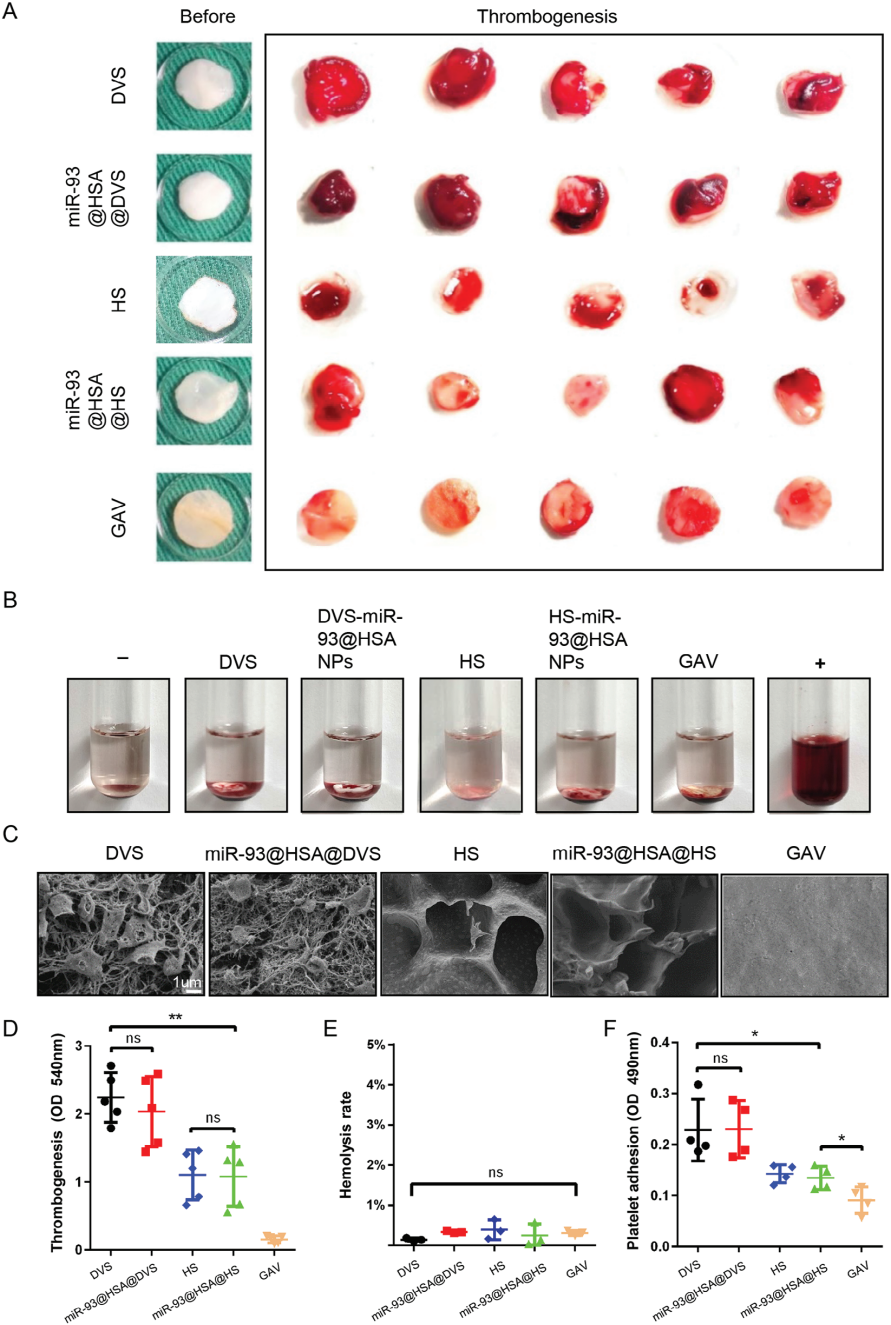
In vitro hemocompatibility of the immune‐modulating scaffold. A) Photographs of thrombogenesis on different scaffolds after incubating with blood. B) Hemolysis rates of the scaffolds. C) Platelet adhesion of the scaffolds observed by SEM. D) Quantification of the thrombus on the scaffolds. *n* = 5. E) Quantification of hemolysis rates of the scaffolds. *n* = 3. F) Quantification of the platelet on the scaffolds. *n* = 4. Data were expressed as mean ± SD, ^*^ *p* < 0.05, ^**^ *p* < 0.01, ^***^ *p *< 0.001. DVS: decellularized valve scaffolds; miR‐93@HSA@DVS: miR‐93@HSA nanoparticles coated DVS; HS: hydrogel coated DVS; miR‐93@HSA@HS: hydrogel and miR‐93@HSA nanoparticles coated DVS; GAV: glutaraldehyde crosslinked aortic valve.

Considering valve working in the hemodynamic environment, material thrombosis remains a crucial challenge in the development of TEHVs. Representative photographs demonstrate that the GAV group exhibited reduced thrombus formation on its surface, whereas both the DVS group and the miR‐93@HSA@DVS group exhibited evident thrombus deposition. Notably, the HS and HS‐miR‐93@HSA‐NPs groups displayed significantly less thrombus formation compared to the DVS group (**Figure** **3**A,D). The hemolysis rates of DVS, miR‐93@HSA@DVS, HS, miR‐93@HSA@HS and GAV scaffolds were measured as 0.145 ± 0.0.024%, 0.335 ± 0.015%, 0.390 ± 0.140%, 0.249 ± 0.164% and 0.31 ± 0.037% respectively. There were no significant differences observed among all groups, and all values were below 5%, indicating neither hydrogel nor NPs modification led to an increase in the hemolysis risks associated with the scaffolds (Figure 3B,E). SEM showed the quantity and morphology of platelets adhering to the surface of scaffolds in each group. The DVS group exhibited a significant presence of platelets, which appeared activated and displayed an irregular, flattened dendritic morphology. Conversely, the GAV group demonstrated minimal platelet adhesion. The miR‐93@HSA@DVS group also showed an increased adhesion of activated platelets, whereas the HS and miR‐93@HSA@HS groups exhibited only a small amount of inactive platelet adhesion (Figure 3C). LDH kit was used to quantify the platelet adhesion of scaffolds in each groups. The results indicated that after modification with hydrogel, HS and miR‐93@HSA@HS groups markedly reduced the number of adhering platelets compared to the DVS group (Figure 3F).

### 2.4 In Vitro Endocytocompatibility and Macrophage Reprogramming of the Immune‐Modulating Scaffold

**Figure 4:**
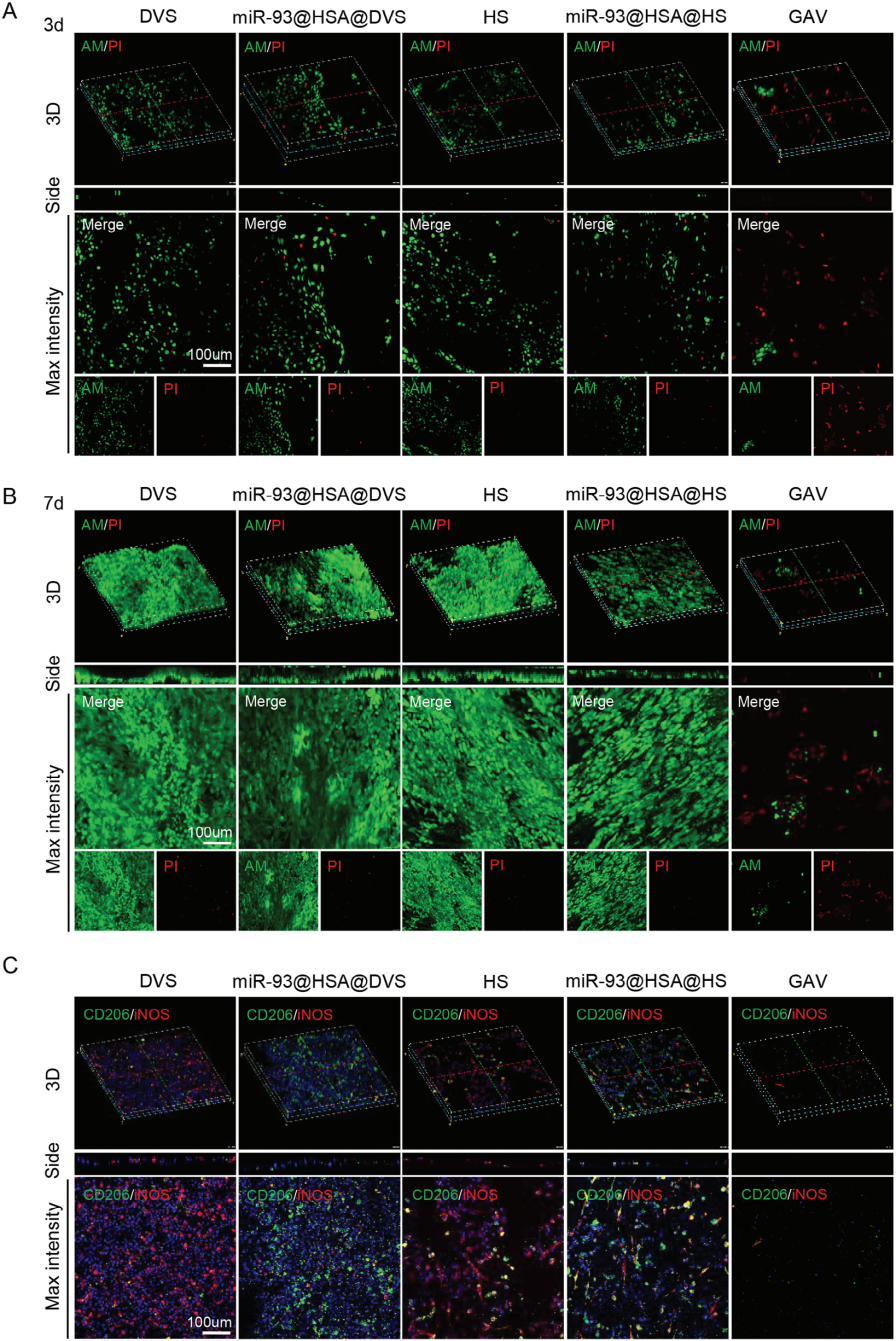
In vitro endocytocompatibility and macrophage reprogramming of the immune‐modulating scaffold. A) Live/dead cell staining of scaffolds seeded with HUVECs after 3 days. B) Live/dead cell staining of scaffolds seeded with HUVECs after 7 days. (live; green), (dead; red). Scale bars = 100 µm. C) Immunofluorescence staining of the BMDMs seeded in different scaffolds. (iNOS; red), (CD206; green). Scale bars = 100 µm. DVS: decellularized valve scaffolds; miR‐93@HSA@DVS: miR‐93@HSA nanoparticles coated DVS; HS: hydrogel coated DVS; miR‐93@HSA@HS: hydrogel and miR‐93@HSA nanoparticles coated DVS; GAV: glutaraldehyde crosslinked aortic valve.

After staining for live and dead cells, confocal microscopy showed that on day 3, there were relatively few endotheliocytes surviving on the scaffold surfaces of each group, distributed as scattered spots. The majority of cells in the GAV group appeared to be dead (**Figure** **4**A). On the 7th day of cell seeding, the maximum intensity of confocal microscope *z*‐axis scanning (Figure 4B, Max intensity) showed that the valve surfaces of the DVS, miR‐93@HSA@DVS, HS, and miR‐93@HSA@HS groups formed a relatively intact layer of endothelial cells, with relatively few dead cells present. In both 3D and lateral views, endothelial cells were observed to grow along the valve surface. The surface of the scaffolds in the DVS and miR‐93@HSA@DVS groups exhibited significant fluctuations, whereas the HS and miR‐93@HSA@HS groups displayed a relatively smoother surface (Figure 4B). CCK8 assay showed that hydrogel‐modified groups (HS and miR‐93@HSA@HS) had faster proliferation during the initial 1–3 days, followed by a slowdown in growth between the 3–7 days, in comparison to the DVS group (Figure S3, Supporting Information). For the immune response between macrophages and biomaterials, mature BMDMs were resuspended and seeded on the surface of the scaffolds in vitro.^[^ ^18^, ^19^ ^]^ In the previous study, BMDMs isolated from SD rats displayed limited activity and adhesion to scaffold walls following the digestive process. Consequently, raw264.7 was selected and IFN‐γ was used to enhance macrophage adhesion and differentiation. After culturing and stimulating the macrophages for five days, fixed staining and confocal microscopy were performed. It was found that there were a large number of macrophages on the surface of DVS, miR‐93@HSA@DVS, HS, and miR‐93@HSA@HS groups, with relatively fewer macrophages found on the GAV group. Furthermore, the majority of macrophages on the DVS and HS groups exhibited characteristics of M1 macrophages, whereas macrophages on the miR‐93@HSA@DVS and miR‐93@HSA@HS groups predominantly displayed an M2 phenotype (Figure 4C).

### 2.5 In Vivo Histocompatibility and Immune Remodeling After Subcutaneous Implantation

**Figure 5:**
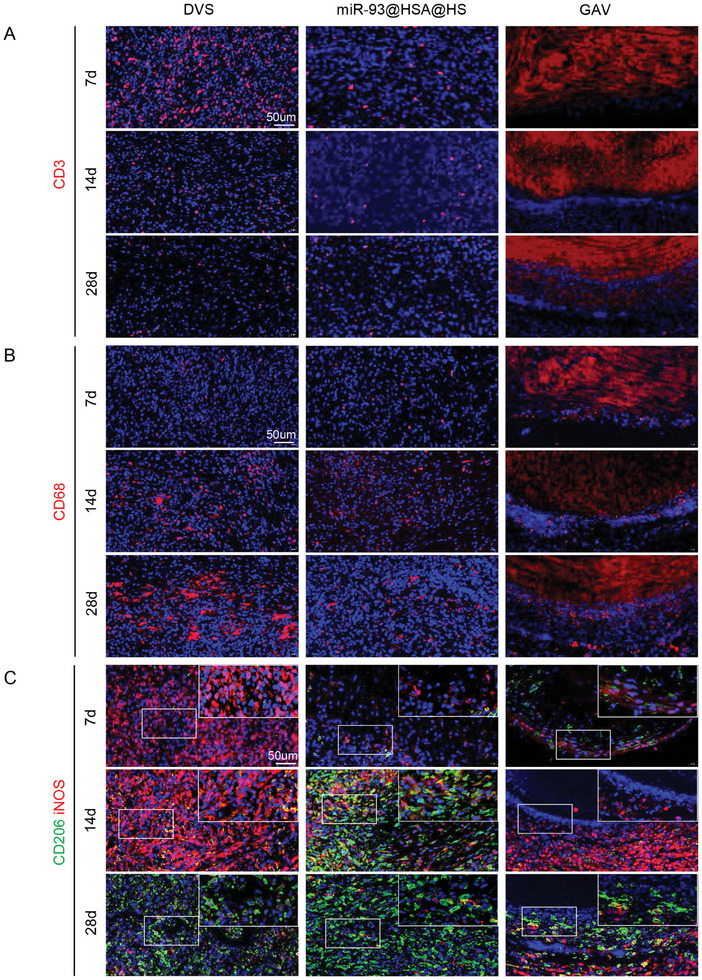
In vivo histocompatibility and immune remodeling after subcutaneous implantation. A) Immunofluorescence staining of CD3+ T cells in scaffolds after 7‐days, 14‐days, and 28‐days implantation. B) Immunofluorescence staining of CD68+ macrophages in scaffolds after 7‐days, 14‐days, and 28‐days implantation. Scale bars = 50 µm. C) Immunofluorescence staining of macrophage subtypes in scaffolds after 7‐days, 14‐days, and 28‐days implantation. iNOS (red) stain for M1 macrophages; CD206 (green) stain for M2 macrophages. DVS: decellularized valve scaffolds; miR‐93@HSA@HS: hydrogel and miR‐93@HSA nanoparticles coated DVS; GAV: glutaraldehyde crosslinked aortic valve.

The rat subdermal model serves as a widely utilized approach for assessing the cytocompatibility, immune response, and tissue remodeling capabilities of scaffolds in vivo.^[^ ^20^, ^21^ ^]^ In this work, three groups of scaffolds (DVS, GAV, miR‐93@HSA@HS) were implanted subcutaneously into rats. As shown in **Figure** **5**A, after subcutaneous implantation for 7 days, cell infiltration in the DVS group was obvious, whereas there was minimal cell infiltration observed in the GAV group. For miR‐93@HSA@HS, the material surface exhibited a dense population of infiltrating cells, but fewer infiltrating cells were present in the interior regions of the scaffold. Masson staining showed that the blue collagen fibers of the scaffolds in all three groups were well‐preserved. After 14 or 28 days of subcutaneous implantation, both DVS and miR‐93@HSA@HS exhibited varying degrees of degradation, whereas the GAV scaffold remained well‐preserved (Figure S4, Supporting Information). From day 7 to 28, there was almost no degradation of collagen fibers and no cell infiltration in the GAV group. Inflammatory responses play a pivotal role in the remodeling process of scaffolds after subcutaneous implantation. At day 7 and 14, the DVS showed a pronounced inflammatory response, with a significantly higher number of CD3+ T cells and CD68+ macrophages compared to the miR‐93@HSA@HS scaffold (Figure 5A,B). In the GAV group, inflammatory cells were primarily confined to the tissue surrounding the scaffolds, with minimal infiltration of CD68+ macrophages or CD3+ T cells within the scaffold itself. At day 28, CD68+ macrophages significantly increased over CD3+ T cells to become the dominant inflammatory cell types in both DVS and miR‐93@HSA@HS groups. To further characterize the phenotype of these macrophages, samples from each group were stained for iNOS (a marker of M1‐type macrophages) and Arg‐1 (a marker of M2‐type macrophages). For DVS, M1 dominated in the scaffolds after 7 or 14‐day subcutaneous implantation; at day 28, there was a shift toward a higher proportion of M2 macrophages (Figure 5C). But for miR‐93@HSA@HS, as early as the 14th day, there was a marked increase in the number of pro‐repair M2 macrophages, which subsequently became the dominant population of macrophages.

### 2.6 Constructive Remodeling of the Immune‐Modulating Scaffold After Abdominal Aortic Implantation

**Figure 6:**
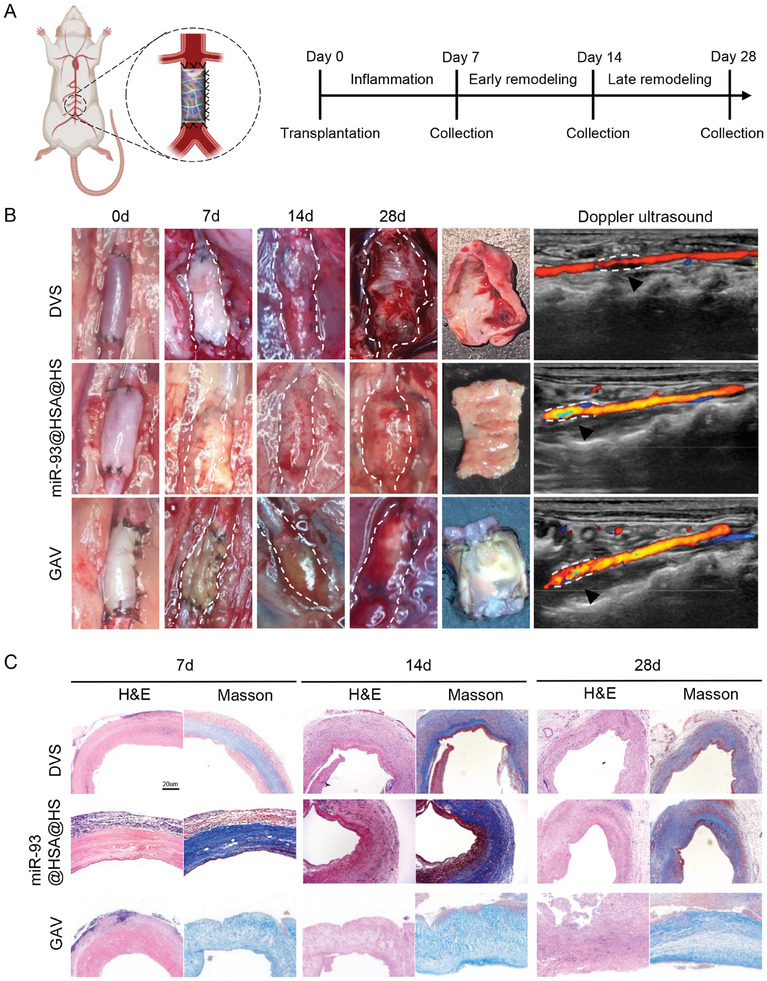
Constructive remodeling of the immune‐modulating scaffold after abdominal aorta implantation. A) Schematic illustration showing the procedure of scaffolds duct‐abdominal aorta heterotopic transplantation. B) General views and ultrasound pictures of the scaffolds after 7‐days, 14‐days, and 28‐days implantation in the rat abdominal aorta. C) Representative histological images of the scaffolds after 7‐days, 14‐days, and 28‐days transplantation. H&E stain indicating cell infiltration of the scaffolds. Masson stain indicating collagen degradation and remodeling. Scale bars = 20 µm. DVS: decellularized valve scaffolds; miR‐93@HSA@HS: hydrogel and miR‐93@HSA nanoparticles coated DVS; GAV: glutaraldehyde crosslinked aortic valve.

Herein, a scaffold duct‐SD rat abdominal aorta heterotopic transplantation model was used to evaluate the performance of scaffolds surrounding the hemodynamic environment (**Figure** **6**A). After 7 days of transplantation, the scaffolds in all three groups exhibited a clear tubular shape with minimal tissue wrapping around their surfaces. At day 14, the DVS displayed slight local bulging or elongation, whereas the diameters of the scaffolds in the GAV and miR‐93@HSA@HS groups remained relatively unchanged. By day 28, the DVS scaffolds exhibited significant dilatation resembling an aneurysm, and while the miR‐93@HSA@HS scaffolds also showed a certain degree of dilatation, the GAV scaffolds maintained their tubular structure. Moreover, the DVS scaffolds observed a thick thrombus attached to the wall, and the GAV scaffolds showed obvious calcification. At day 28, all the scaffolds maintained patency, as confirmed by Doppler ultrasound (Figure 6B). Histological evaluation showed that the degradation and remodeling processes of scaffolds were comparable to those observed in the subcutaneous implantation model (Figure 6C). Compared with DVS, miR‐93@HSA@HS exhibited better‐preserved collagen and smoother intima; different with GAV, which has almost no cell infiltration, miR‐93@HSA@HS showed significant cellular reconstitution.

### 2.7 Dynamic Evolution of Immune Microenvironment and Endothelialization After Abdominal Aorta Implantation

**Figure 7:**
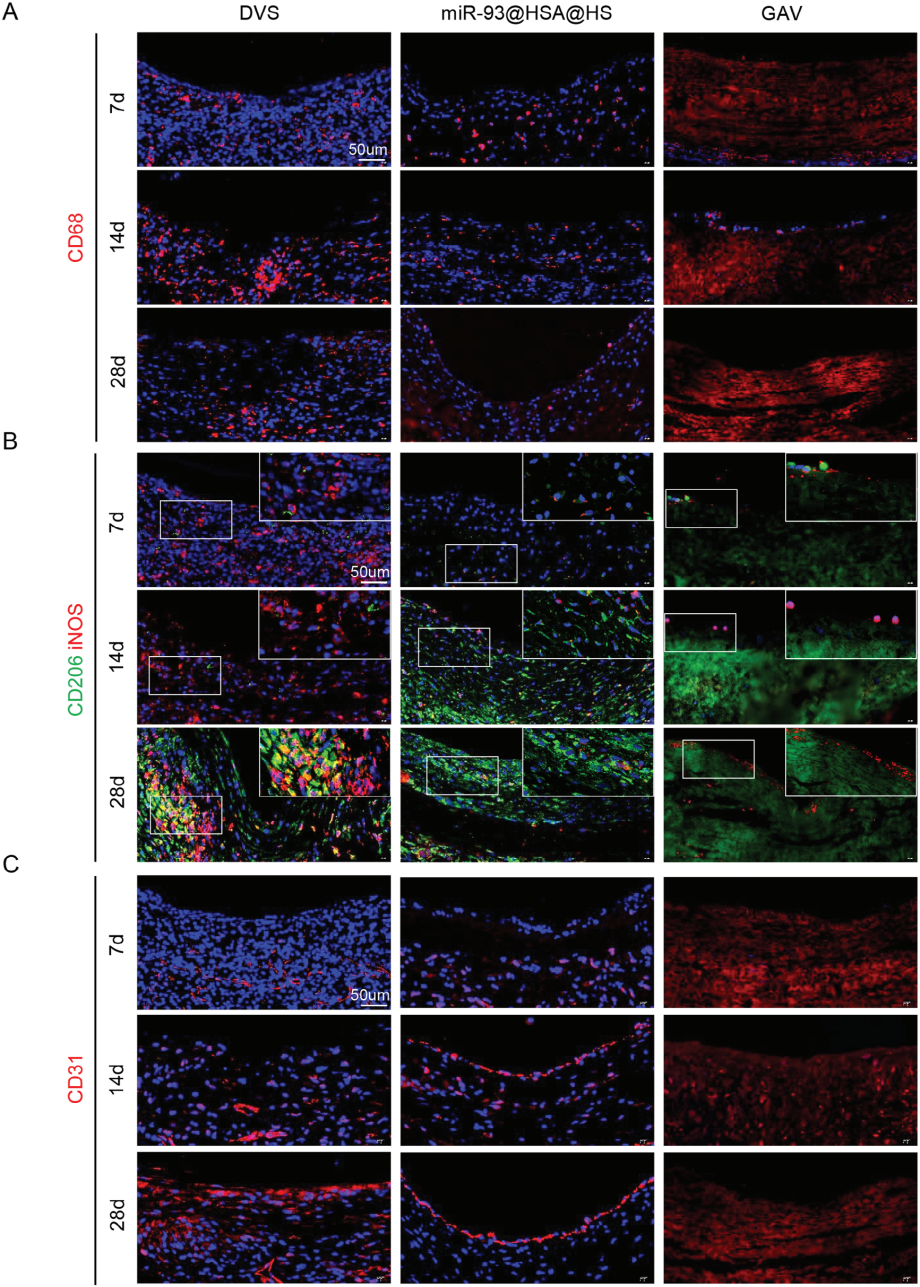
Dynamic evolution of immune microenvironment and endothelialization after abdominal aorta implantation. A) Immunofluorescence staining of macrophages in scaffolds after 7‐days, 14‐days, and 28‐days implantation in the rat abdominal aorta. Scale bars = 50 µm. B) Immunofluorescence staining of macrophage subtypes in scaffolds after 7‐days, 14‐days, and 28‐days implantation in the rat abdominal aorta. iNOS (red) stain for M1 macrophages; CD206 (green) stain for M2 macrophages. Scale bars = 50 µm. C) Immunofluorescence staining of CD31+ endothelial cell in scaffolds after 7‐days, 14‐days, and 28‐days implantation in the rat abdominal aorta. Scale bars = 50 µm. DVS: decellularized valve scaffolds; miR‐93@HSA@HS: hydrogel and miR‐93@HSA nanoparticles coated DVS; GAV: glutaraldehyde crosslinked aortic valve.

In order to assess the dynamic evolution of the immune microenvironment and endothelialization, scaffolds were observed at various time points following abdominal aorta implantation. After 7 days of transplantation, the DVS showed a severe inflammatory response, with a significant increase in CD3+ T cells and CD68+ macrophages. CD3+ cells were more widely distributed throughout the entire scaffold graft (**Figure** **7**A; Figure S5, Supporting Information). At day 14, the number of CD3+ T cells decreased, but the number of CD68+ macrophages increased significantly compared to day 7. At the 28th day, the number of CD3+ T cells had further decreased, and the number of CD68+ macrophages was still high, it did not differ significantly from the count at day 14. Over time, the number of inflammatory cells changes were primarily observed around the scaffolds of the GAV group and not within them. These results indicated that there were more CD3+ T lymphocytes in the DVS and GAV groups at the early stage. However, at day 28, macrophages, particularly M2 macrophages, became dominant, consistent with the findings from the subcutaneous implantation model. Compared to DVS, the inflammatory response to miR‐93@HSA@HS was more moderate, and CD68+ macrophages predominated after 4 weeks of implantation. Further investigation revealed that iNOS+ M1 macrophages were abundant in the scaffolds of both DVS and miR‐93@HSA@HS at the early stage (at 7th day), while Arg‐1+ M2 macrophages dominated at the later stage (at 28th day). A major difference in immune responses to DVS and miR‐93@HSA@HS was the higher proportion of Arg‐1+ macrophages and lower levels of iNOS+ macrophages in miR‐93@HSA@HS at day 14, which could lead to different remodeling processes and promote the formation of an immune‐regulatory microenvironment (Figure 7B).

The endothelialization of scaffold grafts was evaluated with immunofluorescence staining of CD31 (Figure 7C). Post‐transplantation at day 7, a small number of endothelial cells could be detected inside the grafts in the DVS and miR‐93@HSA@HS groups. However, no apparent endothelial cells were visible on the inner lining of the scaffold grafts across all groups. At day 14, endothelial cells in both the DVS and miR‐93@HSA@HS increased significantly. While the endothelial cells in the DVS primarily clustered inside the grafts, a discontinuous linear pattern of endothelial cells was also evident on the inner surface of the miR‐93@HSA@HS grafts. By day 28, a continuous single layer of linear endothelial cells had formed on the surface of both the DVS and miR‐93@HSA@HS grafts, contrasting with the absence of such a layer in the GAV group. Furthermore, the extent of re‐endothelialization was greater in the miR‐93@HSA@HS group.

### 2.8 Mechanism of Immune‐Modulating Scaffold Accelerating Endothelialization

**Figure 8:**
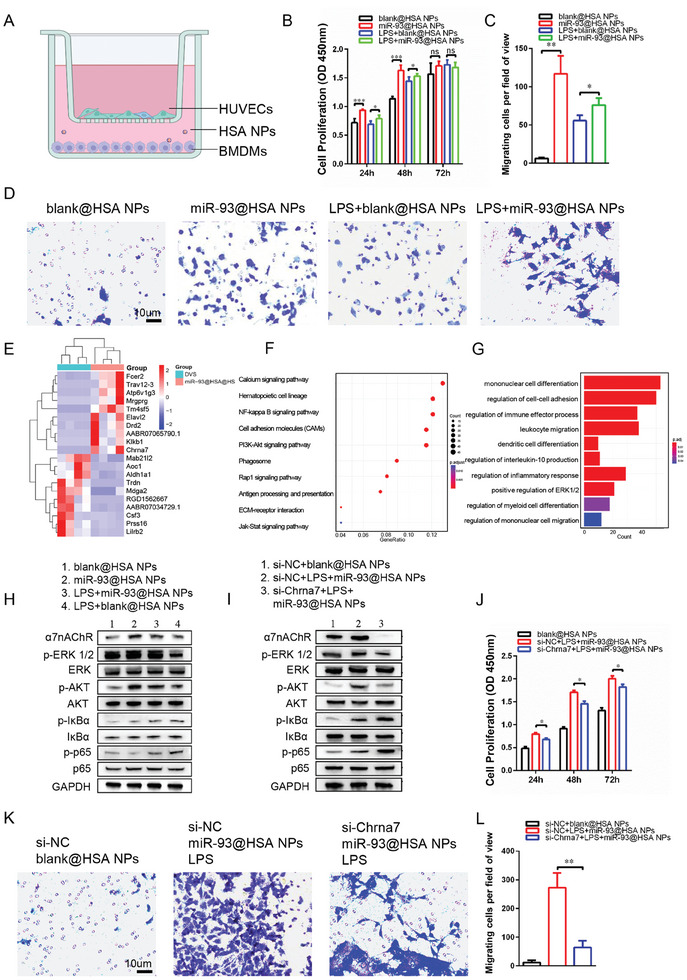
Mechanism of immune‐modulating scaffold accelerating endothelialization. A) The schematic illustration showing the co‐culture of BMDMs and HUVECs. B) HUVECs proliferation detected with CCK‐8. *n* = 6. C,D) The migration of HUVECs was detected with transwell assay. *n* = 3. E–G) RNA‐sequence results of scaffolds after 14‐days implantation in the rat abdominal aorta. E) Heatmap of the top twenty differentially expressed genes, sorted by log fold change. F) Gene ontology terms enrich the highly expressed genes of the DVS group versus miR‐93@HSA@HS groups. G) KEGG Pathway enriches group DVS versus group miR‐93@HSA@HS highly expressed genes. H,I) The protein expression level of α7nAChR, ERK, AKT, IκBα, p65. J) HUVECs proliferation detected with CCK‐8. K,L) The migration of HUVECs detected with transwell assay. Data were expressed as mean ± SD, ^*^ *p *< 0.05, ^**^ *p *< 0.01, ^***^ *p *< 0.001.

The mechanism of immune‐modulating scaffold accelerating endothelialization is to elicit. As depicted in the schematic illustration, a transwell device was used to be as the cell co‐culture system, with HUVECs placed in the upper chamber and BMDMs in the lower chamber (**Figure** **8**A). The results suggest that macrophage after miR‐93@HSA NPs treatment significantly promotes migration of HUVECs, regardless of whether LPS and IFN‐γ intervention is present (Figure 8C,D). Additionally, the proliferation capacity of HUVECs was enhanced by macrophage supernatant after miR‐93@HSA NPs treatment (Figure 8B).

The constructive remodeling of scaffolds in miR‐93@HSA@HS group was closely associated with a reduced presence of M1‐type macrophages and a significantly higher proportion of M2‐type macrophages.^[^ ^22^ ^]^ Macrophage polarization triggered by scaffolds is an intricate process.^[^ ^23^ ^]^ Given that early endothelialization occurred on day 14, specimens from both the DVS and miR‐93@HSA@HS groups were collected after 14 days of abdominal aortic implantation for transcriptome sequencing. The results revealed that nicotinic acetylcholine receptor alpha7 subunit (Chrna7) was significantly increased in group miR‐93@HSA@HS (Figure 8E). Gene ontology and KEGG Pathway enrich NFκB/p65 pathway and ERK1/2 cascade. (Figure 8F,G). The level of α7nAChR, p‐IκBα, and p‐p65 was significantly increased in HUVECs cultured with macrophage supernatant after miR‐93@HSA NPs treatment (Figure 8H). And the expression of α7nAChR, p‐ERK 1/2, p‐AKT, p‐IκBα, and p‐p65 was depressed in HUVECs with Chrna7 knockdown cultured in macrophage supernatant after miR‐93@HSA NPs treatment (Figure 8I). Moreover, Chrna7 knockdown also attenuated both the proliferation and migration of HUVECs (Figure 8J–L).

## 3 Discussion

TEHV, with the ability to grow and repair itself, stands as an ideal valve substitution. Emerging evidence now suggests that in situ regeneration is a simple and efficient method to make TEHV, with an optimal scaffold serving as its cornerstone. But scaffolds, no matter how perfect they are, are prone to persistent inflammation. Our approach aims to harness this double‐edged sword of inflammation, capitalizing on its endothelialization‐promoting capabilities while mitigating the damaging effects of inflammation, ultimately leading to constructive remodeling. The major strategies based on the immune‐modulating theory are as follows: i) Galactose‐modified nanoparticles deliver miR‐93 mimics, specifically targeting macrophages and orchestrating their differentiation toward the M2 phenotype. This process creates a beneficial immune microenvironment. ii) MMP‐responsive hydrogel is used to encapsulate these nanoparticles, enabling targeted delivery and sustained release. This immune‐modulating scaffold promises to mitigate early inflammatory damage and provide a pro‐endothelialization niche, crucial for the constructive remodeling of TEHVs.

Biomaterials, upon implantation within the body, can elicit varying degrees of innate and adaptive immune responses. This cascade commences with an acute inflammatory reaction, which, in certain instances, may escalate into chronic inflammation, a foreign body response, and ultimately, fibrous encapsulation. The intensity and longevity of this inflammatory process directly affect the function and stability of the material.^[^ ^24^ ^]^ After the implantation of biological valve materials, inflammation mediates the recruitment and infiltration of monocytes, macrophages, dendritic cells, and neutrophils; and these immune cells secrete IL‐6, IL‐1β, TNF‐α and other pro‐inflammatory factors, resulting in local tissue damage. In the later stage, these infiltrating immune cells undergo phenotypic shifts and release anti‐inflammatory and repair factors, such as IL‐10, Arg‐1, VEGF, IGF‐1, etc.; and these factors contribute to the remodeling of valve materials.^[^ ^23^ ^]^ In the ideal case, a process known as “constructive remodeling” ensues. This involves the orchestration of repair factors that stimulate the formation of novel extracellular matrix. Furthermore, it prompts the migration, proliferation, and differentiation of endothelial progenitor cells and mesenchymal stem cells into valve endothelial cells and interstitial cells, respectively.

To construct this immune‐modulating TEHV, the extracellular matrix obtained from natural aortic valves after acellular treatment was used as a basement. DVS completely preserved the original shape and spatial structure of the valve, providing a good environment for in situ reendothelialization.^[^ ^25^ ^]^ In line with the principle of removing only xenogeneic cells and preserving the extracellular matrix of the natural valve as well as possible, this study was based on the continuous proteolytic method previously explored by our research group to prepare acellular valves.^[^ ^26^ ^]^ However, once cells are removed, extracellular matrix is completely exposed to the blood environment and faces the impact of various components in the blood. In the absence of endothelial protection, platelets and plasma proteins are easy to adhere to bare scaffolds, leading to thrombosis.^[^ ^27^ ^]^ To improve the hemodynamic performance of scaffolds, hydrogels were used to encapsulate DVS, safeguarding the extracellular matrix during the initial in vivo implantation phase. Hydrogels can be broadly categorized into natural and synthetic types. The main synthetic hydrogels used in heart valves are polyethylene glycol (PEG) hydrogels and polyvinyl alcohol (PVA) hydrogels.^[^ ^28^ ^]^ While PEG hydrogels excel in biocompatibility and non‐immunogenicity, making them popular in tissue engineering, they may not readily support cell adhesion and growth in a blood flow environment. In previous studies, peptides such as RGD peptide are often added to facilitate cell adhesion and proliferation.^[^ ^29^, ^30^ ^]^ Natural hydrogels exhibit good biocompatibility and bioactivity mainly composed of substances found in natural extracellular matrix, such as collagen, hyaluronic acid, and sodium alginate. In this study, OSA and amino gelatin were used to prepare double‐crosslinked hydrogels through a unique blend of chemical and optical crosslinking techniques. During the plasm protein adhesion phase, this newly‐constructed TEHV scaffold decreases thrombosis formation by providing a relatively flat surface, where the OSA‐MMP/AG hydrogel encapsulation of DVS minimizes protein adhesion.

To help modify OSA‐_MMP_/AG hydrogel to the DVS scaffold, maleimide was introduced into the DVS, enabling it to react with the sulfydryl groups of the MMP substrate peptide. The mechanism of slow release of miR‐93 mimics is that circulating monocytes derived macrophages infiltrate into scaffolds and release MMPs, which degrade the OSA‐conjugating peptide and hydrogel, exposing the miR‐93@HSA NPs on the scaffold surface, which are then engulfed by the macrophages.^[^ ^31^ ^]^ During the inflammatory phase, this newly‐constructed valve scaffold provides a pro‐endothelialization niche by in‐situ conditional release of miR‐93@HSA NPs, which can reprogram macrophages from a pro‐inflammatory to an anti‐inflammatory phenotype. At proliferative stage, this M2‐phenotype macrophage, which secretes a large number of anti‐inflammatory factors such as VEGF and IGF‐1, IL‐10, TGF‐β, facilitates the formation of a new blood vessel and promote the deposition of collagens.^[^ ^32^ ^]^ Crosstalk between immune and endothelialization is crucial for transitioning from the inflammatory stage to matrix remodeling and endothelial formation. The α7nAChR, as an important role of “cholinergic anti‐inflammatory pathway”, is required for inhibition of M1‐related cytokines release.^[^ ^33^ ^]^ Activating α7nAChRs promotes angiogenesis and endothelial repair via enhancing endothelial progenitor cells functions.^[^ ^34^ ^]^ In this study, transcriptome sequencing also revealed α7nAChR was significantly increased in group miR‐93@HSA@HS. The α7nAChR may be a bridge linking macrophages and endothelia cells, eliciting the role of immune‐modulating scaffold accelerating endothelialization.

The seed cells used for in situ endothelialization of TEHVs are mainly composed of adjacent endothelial migration or circulating stem cell migration and colonization to the surface of scaffolds. In either case, good endothelial compatibility of scaffolds is required.^[^ ^35^ ^]^ Implantation of specific cells on the surface of scaffolds is a common method for TEHVs to be constructed in vitro or evaluated for cytocompatibility.^[^ ^36^ ^]^ CCK8 kit was used to detect the cell activity of HUVECs implanted on the surface of the scaffold at day 1, 3 or 7. Results showed that the number of living cells on the scaffolds in each group increased with the extension of time within a week. It was also found that the proliferation of MMP‐responsive hydrogel encapsulated DVS groups, namely HS and miR‐93@HSA@HS groups, was faster in the early 1–3 days, but slowed down in the 3–7 days compared with the DVS group. This may be directly related to the modification of DVS by MMP‐responsive hydrogels. Due to the encapsulation of hydrogels, the originally uneven surface of DVS becomes relatively flat. During in vitro implantation, HUVECs are more likely to stick to the wall for growth, so the early proliferation is relatively fast. Over time, hydrogel occurred slight swelling in the medium, which may be related to the slowing down of cell proliferation in HS and miR‐93@HSA@HS groups.

Considering the work environment of heart valves is circulating blood, a scaffold duct‐SD rat abdominal aorta heterotopic transplantation model was used to evaluate the performance of scaffolds surrounding the hemodynamic environment. First, the scaffold was rolled into a pipe matching the rat abdominal aorta, and then was anastomosed end to end with the severed rat abdominal aorta to observe the status of the valve material in the blood flow. At 28 days, ultrasound was used to detect the patency of the graft valve pipeline in each group, and it was found that the blood flow in the valve material pipeline in the DVS, GAV, and miR‐93@HSA@HS groups remained patency. In addition, the grafts of the DVS group were obviously dilated and the blood flow status was changed to some extent.

The results indicated that at the initial stage, the DVS and GAV groups exhibited a higher presence of CD3+ T lymphocytes. However, as the experiment progressed to day 28, macrophages, particularly M2 macrophages, became the predominant cell type, echoing the observations made during subcutaneous implantation. In contrast, the miR‐93@HSA@HS group, due to hydrogel coating, CD3+, and CD68+ cells were few in the early stage. By day 28, CD68+ macrophages emerged as the dominant cell type. Intriguingly, at day 14, the count of M2 macrophages surpassed that of M1 macrophages, and this trend became increasingly pronounced by day 28^[^ ^37^ ^]^. In addition to the effect of miR‐93@HSA NPs, surface characteristics such as hydrophilicity and hydrophobicity of scaffolds will also affect the phenotype of macrophages, which may be further explored in subsequent work.^[^ ^38^, ^39^ ^]^ CD31 fluorescence staining of endothelial showed that only a few endothelial could be seen in the matrix of DVS and miR‐93@HSA@HS groups at day 7. At day 14, endothelial in the inside matric of DVS increased significantly, but no endothelial was found on the lumen surface, while some discontinuous linear endothelial were found on the lumen surface in the miR‐93@HSA@HS group. At day 28, the single layer of endothelial was observed on the lumen surface of scaffolds in the DVS and miR‐93@HSA@HS groups, but the single layer of endothelial on the lumen surface of the graft was more complete in the miR‐93@HSA@HS group. However, the abdominal aorta model is not insufficient for reflecting the heart valve conditions. We sutured miR‐93@HSA@HS into transcatheter heart valves for in vitro pulsatile flow testing to assess mechanical properties, with DVS and GAV as controls (Figure S8A,B, Supporting Information). miR‐93@HSA@HS group met the criterion of cardiac valve prostheses (ISO 5840), which prescribes a minimum effective orifice area of 1.45 cm^2^ and a maximum regurgitant flow rate of 20%.

## 4 Conclusion

In this study, miR‐93@HSA NPs were employed to modify the DVS, whereas an MMP‐responsive hydrogel served to encapsulate the nanoparticles, facilitating targeted delivery and sustained release. This innovative scaffold effectively prompted the polarization of macrophages toward the M2 phenotype, thus regulating the immune response triggered by the implantation of valve material in vivo and accelerating the re‐endothelialization of the valve scaffolds. Our findings suggest that these immune‐modulating scaffolds possess remarkable remodeling capabilities, hinting at their vast potential for use as prosthetic valves in the future.

## 5 Experimental Section

### Materials

The four‐armed poly(ethylene glycol) macromer featuring maleimide groups at both ends (PEG‐4MAL, 20 kDa, purity > 95%), galactose, HSA, and polyethyleneimine (PEI) were obtained from the Xi'an ruixi Biological Technology Co., Ltd. (Xi'an, China). The miRNA‐93‐5p mimic was purchased from the Suzhou GenePharma Co., Ltd. (Suzhou, China). MMP degradable peptide (Ac‐GCRD‐GPQG‐IWGQ‐DGCG‐NH2, 1.7 kDa) was obtained from the Shanghai GL Biochem Ltd. (Shanghai, China). Sodium alginate (SA, CAS number: 9005‐38‐3) and gelatin (CAS number: 9000‐70‐8) were purchased from Dalian Meilunbio Co., Ltd. (Dalian, China). Chemicals relevant to decellularization were obtained from Sigma Aldrich (St. Louis, MO, USA). CCK‐8 Kit was obtained from Dojindo Molecular Technologies. The lactate dehydrogenase (LDH) Cytotoxic Assay Kit was purchased from Cayman Chemical Company. LIVE/DEAD Assay Kits were purchased from Life Technologies. Primary antibody and fluorescent secondary antibody were purchased from BD. Fetal bovine serum (FBS), phosphate buffer saline (PBS) and Dulbecco‐modified Eagle medium (DMEM) were purchased from Gibco.

### Animals

Male Sprague‐Dawley (SD) rats (from Charles River Laboratory Animal Technology Co., Ltd, Beijing) were used. All these animal experiments in this study were conducted strictly according to the Guide of Care and Use of Laboratory Animals, approved with the Laboratory Animal Ethics Committee of Tongji Medical College, Huazhong University of Science and Technology (IACUC number: 3288).

### Bone Marrow‐Derived Macrophage Isolation and Culture

Bone marrow‐derived macrophages (BMDMs) were isolated from SD rats (male, 60–80 g), as described previously. Specifically, SD rats were euthanized using cervical dislocation. The tibia and femur were free from the surrounding muscle and fat tissue, sterilized in 70% ethanol for 10 s, and then washed in PBS. With the ends of each tibia and femur removed, the bone marrow was flushed out by RPM1640 supplemented using a 25‐gauge needle. Next, cells from the bone marrow were passed through a cell strainer of 70um and washed with PBS after centrifugation (500 g, 3 min). Then, these cells were plated in two dish of 10 cm at about 13000 cells/8 mL media that were composited by RPM1640 with 10% FBS, M‐CSF 10 ng mL^−1^, 1% penicillin/streptomycin. With BMDMs grown at 37 °C for 7 days, the media was removed and replaced with the same fresh media every 2 days. Flow cytometry showed the purity of macrophages at about 98% at 1 week. At the 7th day, BMDMs were treated for 6 h with different treatments: control (no treatment); LPS group (LPS 100 ng mL^−1^); miRNA group (LPS 100 ng mL^−1^ and miRNA mimic 200 µm); IL‐4 group (IL‐4 10 ng mL^−1^).

### Preparation of Nanoparticles Containing miRNA Mimics

PEG‐HSA nanoparticle targeting macrophage was prepared via the layer by layer self‐assembly technology. Briefly, miRNA mimics or miRNA scramble control, galactose, and PEG‐HSA were all dissolved in the deionized distilled water, respectively. Firstly, miRNA mimics solution was mixed with cross‐linked PEI (cPEI) at a weight/weight of 4 (the ratio of weight of miRNA mimics to cPEI) and incubated for 15 min at 25 °C. Next, galactose of different concentration gradients and the solution of PEG‐HSA were mixed with gently stirred and incubated at 25 °C for 30 min. The mixture of miRNA mimics and cPEI was premixed with the mixture of galactose and PEG‐HSA absolutely. Then, the mixed coarse suspension of these two mixtures was added drop by drop into buffer solution (PBS, 0.1 m, pH 7.4) with a gently stir. Lastly, glutaraldehyde was used to cross‐link the resulting solution (overnight, 25 °C).

### Characterization of Nanoparticles Containing miRNA Mimics

Particle size and zeta potential were assessed through NanoZS. SEM (Zesis) was used to show the morphology of the PEG‐HSA nanoparticle. And the multimode microplate reader (TECAN Infinite M200 PRO) was used to detect the encapsulation efficiency (EF%) and drug loading capacity (DL%).

(1) $$EF\%was\;calculated\;as:\left[\left(Wmir-Wsup\right)/Wmir\right]\times 100\%$$

(2) $$DL\%was\;calculated\;as:\left[\left(Wmir-Wsup\right)/Wt\right]\times 100\%$$

Wmir means the weight of added miRNA mimics, Wsup means the weight of miRNA mimics in the supernatant, and Wt means the total PEG‐HSA nanoparticle weight.

### Cell Transfection with miRNA Mimics

BMDMs were obtained and seeded into six‐well plates with RPMI1640 complete medium (containing 10% fetal bovine serum). After starvation, the miR‐93‐5p mimics or miR‐93‐5p scramble control was transfected to BMDMs with Lipofectamine 3000, respectively.

### Preparation of Hydrogel—Preparation of OSA

Deionized water of 400 mL was put into a large beaker, 5 g SA with medium viscosity was added, heated, and stirred to completely dissolve it. Then, aqueous NaIO_4_ solution with different concentrations was added, and the reaction was stirred at room temperature in the dark for 12 h, then ethylene glycol was added and stirred for 15 min to terminate the reaction. Then the obtained product was put into a dialysis bag (cut off Mw = 14000) and dialyzed in the dark for 3 days (changing water every 6–8 h). After complete dialysis, the product was lyophilized in the lyophilizer to obtain the product for use.

### Preparation of Hydrogel—MMP Grafting

OSA of 2 g was weighed and dissolved in 200 mL of deionized water, stirred, and heated until completely dissolved, then 50 mg MMP substrate peptide was added, and the reaction was carried out at room temperature in the dark for 24 h. Then the obtained product was loaded into a dialysis bag and lyophilized.

### Preparation of Hydrogel—Amino‐Gelatin Preparation

Gelatin of 5 g was accurately weighed and stirred to dissolve in phosphate buffer solution (PH = 5.0), then 8 mL ethylenediamine was added at the molar ratio of ─COOH/ ethylenediamine 1:2 while stirring, and then a certain amount of EDC was added and stirred at room temperature for 8 h. The resulting solution was placed in a dialysis bag for dialysis for 3 days (changing water every 6–8 h). After the analysis is complete, freeze–dry in the lyophilizer to obtain the product for use.

### Preparation of Hydrogel—Decellularized Valve Hydroxylamine Hydrochloride Block Carboxyl Group

The decellularized porcine aortic valve was sealed in 1 m hydroxylamine hydrochloride at room temperature. After sealing, it was rinsed with distilled water and stored.

### Preparation of Hydrogel—Mercaptoylation of Decellularized Valves

The decellularized porcine aortic valves were immersed in PBS (0.1 m, PH 7.4, containing 1 mm EDTA) at room temperature, and 2 mg mL^−1^ SATA was added at 25 °C, 1000 rpm for 2 h. The reaction was terminated by washing with PBS and 0.5 m hydroxylamine hydrochloride solution, PH 7.5, was added at 25 °C for 2 h.

### Preparation of Hydrogel—Adsorption of Nanoparticles by Sulfhydrylated Decellularized Valves

The sulfhydrylated valves were freeze–dried and mixed with the nanoparticle solution for adsorption, and shaken at room temperature for 24 h.

### Preparation of Hydrogel—Hydrogel and Adsorbed Nanoparticles Acellular Valve Composite

The aqueous solution of 20% (w/v) MMP‐ASA and amino‐gelatin was mixed, and the photoinitiator I2959 (the final concentration of I2959 was 0.1% w/v) was added to stir well, then the evenly mixed solution was carefully applied to the ventricular surface of the decellularized valve, and the reaction was carried out in a 37 °C incubator for 15 min. Then, the ventricular surface cross‐linked network was obtained by UV irradiation (1 mW cm^−2^) for 15 min, and then the aortic surface hydrogel composite was repeated. Finally, the hydrogel with adsorbed nanoparticles acellular valve composite was obtained.

### Preparation of DVS

According to our previous study, porcine aortic valve leaflets were decellularized with detergent. Briefly describing, after washing away blood, valve leaflets immerse with M3 solution (CHAPS of 2% w/v and TnBP of 2 mm), M4 solution (CHAPS of 2% w/v, TnBP of 2 mm, amidosulfobetaine‐14 of 1% w/v and sulfobetaine 3–10 of 2% w/v) and Benzonase solution (Benzonase Nuclease of 100 unit/mL, MgCl_2_ of 1 mm and NaCl of 1 mm) successively. All condition is 37 °C and 110 rpm. The gotten DAV would be assessed decellularization effect via hematoxylin & eosin (H&E), elastin van gieson (EVG), and masson trichrome staining.

### Proliferation and Viability Assay of HUVECs in Complex Materials

Scaffolds were trimmed into small round pieces adapted to the well of the 96‐well plate. HUVEC (1 × 10^6^ cells mL^−1^) were resuspended and cultured on the scaffolds in the 96‐well plate. CCK‐8 kit (Biosharp, China) was used to detect cell proliferation and draw the curve at every time point (1, 3, 7 days). At day 3 or 7, morphology of dead or alive cell in scaffolds was observed under confocal microscope (Olympus, Japan) after incubation with Calcein AM/PI Cell Viability Assay Kit.

### Thrombogenicity Assay

Scaffolds were trimmed into small round pieces and incubated with the blood of SD rat reconstituted by CaCl_2_ (100 mm) solution (37 °C, 30 min). After washed with PBS, the samples were incubated with 0.5% Triton‐100 for 30 min. Collected supernatant was measured at the wave length of 540 nm.

### Adhesion Assay of Platelets

Platelet‐rich plasma (PRP) was collected from the blood of SD rat, which was rested (10 min) and centrifuged (1500 rpm, 20 min). Scaffolds were incubated with PRP solution (37 °C, 1 h). For quantification, after washed with PBS, samples were incubated with 0.5% Triton‐100 for 30 min. And collected supernatant was detected by the kit of LDH assay (Beyotime Biothchnology).

### Hemolysis Rate Assay

Citrated blood of the SD rat was washed 5 times with normal saline until the supernatant was transparent. Red blood cells (RBCs) were collected after centrifuging at 4000 rpm for 10 min. Samples including scaffolds, negative and positive control were respectively incubated with RBCs solution consisting of normal saline and RBCs at 37 °C for 1 h. The collected supernatant of each sample was measured at 540 nm by a microplate reader. The hemolysis rate was calculated as:

(3) $$\begin{array}{ccc} & & \left(OD_{scaffold\;group}-OD_{negative\;control}\right)\\ & & /\left(OD_{positive\;control}-OD_{negative\;control}\right)\times 100\% \end{array}$$

### In Vivo Assessment by the Model of Subcutaneous Implantation

Models of subcutaneous implantation were constructed according to our previous study. Briefly, SD rats of 80 g were divided into three groups (DVS, glutaraldehyde crosslinked aortic valve (GAV), hydrogel, and miR‐93@HSA nanoparticles coated DVS (miR‐93@HSA@HS)) randomly. After anesthetizing with isoflurane inhalation, the rat abdominal skin was prepared, and made a small incision. The scaffold was put into the subcutaneous space obtained with blunt separation. After 7, 14 or 28 days, scaffolds were collected and characterized by HE, Masson, and immunofluorescent stain to show ECM change, histocompatibility, and immune remodeling.

### In Vivo Assessment by the Model of Abdominal Aorta Implantation

The model of rat abdominal aorta implantation was used to evaluate the performance of the scaffolds (DVS, GAV, miR‐93@HSA@HS) under the hemodynamic environment. After anesthetizing the rats, an incision was made along the linea alba of the abdominal skin. Abdominal tissue was bluntly separated, and the abdominal aorta was fully exposed and separated from a vein. The proximal and distal ends of the free abdominal aorta were carefully clamped with parallel clips, and then the abdominal aorta was cut. The 8‐0 prolene sutures were used to make end‐to‐end anastomosis of the scaffold duct and abdominal aorta. After the anastomosis was completed, aortic clips were opened and the abdomen was closed layer by layer. After 7, 14 or 28 days, scaffolds were collected and characterized by HE, Masson, and immunofluorescent stain to show ECM change, histocompatibility, immune remodeling, and endothelialization.

### Flow Cytometry

The proportion of M1‐type or M2‐type macrophage was assessed with flow cytometry. Treated BMDMs were collected and stained with PE‐CY7‐anti‐CD86 and APC‐anti‐CD206 according to the protocol. Flow cytometry was performed with the FACSCalibur (BD Immunocytometry Systems). And the data was analyzed by FlowJo software.

### Immunofluorescence Stain

Samples of scaffolds or cells were carefully collected and fixed in 4% paraformaldehyde for immunofluorescence stain. The primary antibodies used in immunofluorescence were iNOS (Mouse monoclonal, ab210823, Abcam), Arg‐1 (Rabbit polyclonal, 16001‐1‐AP, Proteintech), F4/80 (Rabbit monoclonal, ab300421, Abcam), CD31 (Rabbit polyclonal, ab32457, Abcam), CD3 (Rabbit monoclonal, 78588, CST), CD68 (Rabbit monoclonal, 76437, CST), and CD206 (Rabbit monoclonal, 91992, CST). The nuclei were stained by DAPI.

### Western Blot

Specimens of raw264.7 were lysed in RIPA solution added with inhibitors of proteinase and phosphatase. BCA assay (Beyotime) was used to make quantification of protein. Extracts of proteins were separated on gels, and transferred to the PVDF membrane. This membrane was washed with TBST buffer, blocked with nonfat milk, and incubated overnight with primary antibodies at 4 °C and secondary antibody for 1 h at room temperature. After being washed with TBST and incubated with ECL kit, bands were detected with a chemiluminescence system.

### Statistics

All data are presented as mean ± standard deviation. An unpaired, two‐tailed Student *t*‐test was used to analyze normally distributed data from two groups. Multiple comparisons were analyzed by one‐way or two‐way analysis of variance (ANOVA). Differences with *p *< 0.05 were considered statistically significant. All the experiments were repeated for at least three replicates per condition.

## Conflict of Interest

The authors declare no conflict of interest.

## Author Contributions

X.C. and Y.W. contributed equally to this work. X.C. and Y.W. performed methodology, investigation, wrote and prepared the original draft. X.C., Y.W., L.F., and P.S. performed methodology, validation, and data curation. Y.Z., J.S. performed formal analysis, Software. W.Q. and N.D. performed formal analysis, and conceptualization. N.D. performed conceptualization, and supervised the project.

## Supporting information



Supporting Information

## Data Availability

The data that support the findings of this study are available from the corresponding author upon reasonable request.
