# Supplementary material for: Matrix Metalloproteinase‐Responsive Controlled Release of Self‐Assembly Nanoparticles Accelerates Heart Valve Regeneration In Situ by Orchestrating Immunomodulation
Source: Adv Sci (Weinh). 2024 Nov 13;12(2):2403351. doi: 10.1002/advs.202403351 (PMC11727384; doi:10.1002/advs.202403351)
Supplement: Supplementary file 1 — Supporting Information [file ADVS-12-2403351-s001.docx]

**Matrix Metalloproteinase-Responsive Controlled Release of Self-Assembly Nanoparticles Accelerates Heart Valve Regeneration in Situ by Orchestrating Immunomodulation**

Xing Chen^1,2#^, Yunlong Wu^1#^, Peng Song^3^, Liandong Feng^4^, Ying Zhou^1^, Jiawei Shi^1^, Nianguo Dong^1*^, Weihua Qiao^1*^

^1^Department of Cardiovascular Surgery, Union Hospital, Tongji Medical College, Huazhong University of Science and Technology, Wuhan 430022, China.

^2^Department of Cardiovascular Surgery, Zhongnan Hospital, Wuhan University, Wuhan 430071, Hubei, China.

^3^School of Chemistry and Engineering, Huazhong University of Science and Technology, Wuhan 430074, Hubei, China.

^4^Hubei Provincial Key Laboratory of Occurrence and Intervention of Rheumatic Diseases, Minda Hospital of Hubei Minzu University, Enshi, China.

^*^Corresponding authors:

Dr. Weihua Qiao, Department of Cardiovascular Surgery, Union Hospital, Tongji Medical College, Huazhong University of Science and Technology, Wuhan 430022, China.

Email: weihua_qiao@hust.edu.cn

Prof. Nianguo Dong, Department of Cardiovascular Surgery, Union Hospital, Tongji Medical College, Huazhong University of Science and Technology, Wuhan 430022, China.

Email: dongnianguo@hotmail.com

^#^ Xing Chen and Yunlong Wu contributed equally to this work.

**
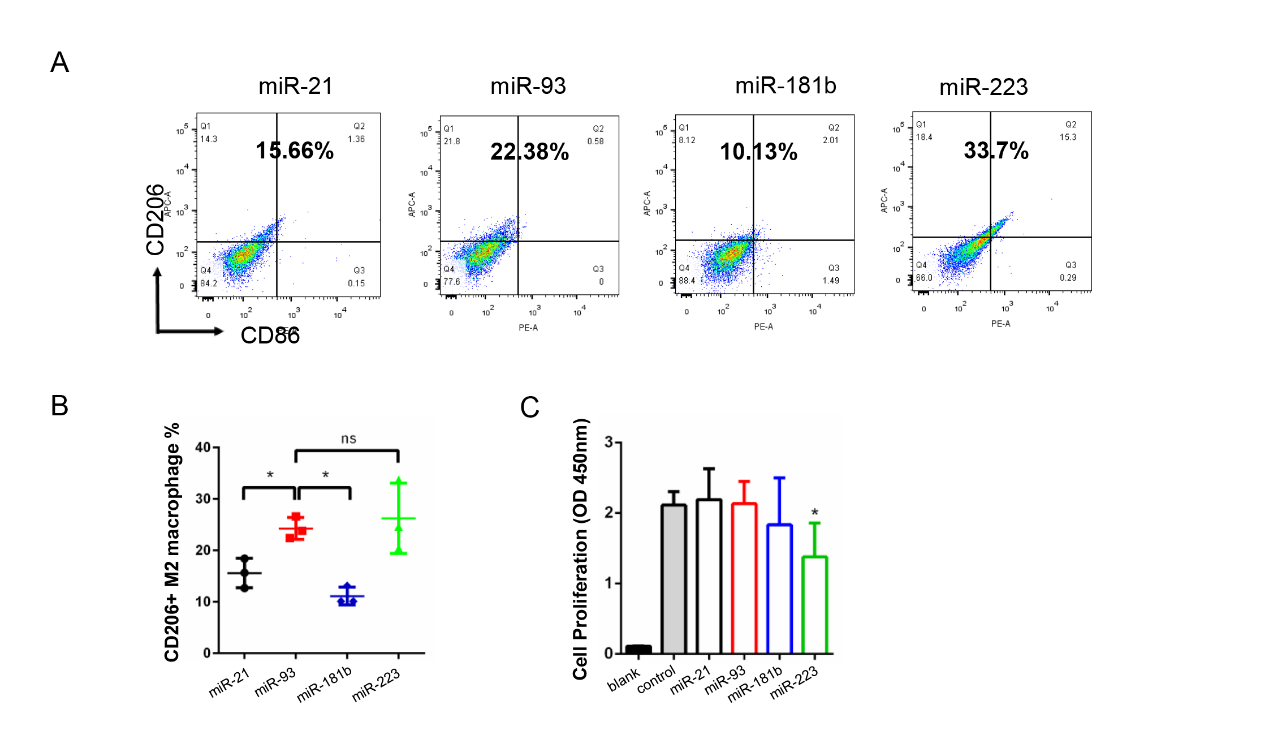
**

**Figure S1. Detection of the M2 polarization ability and effect on the proliferation of endothelial cells.** (A and B) Representative graphs of CD206+ M2 macrophages. n=3. (C) HUVECs proliferation detected with CCK-8. Data were expressed as mean ± SD, *P<0.05, **P<0.01, ***P<0.001.

**
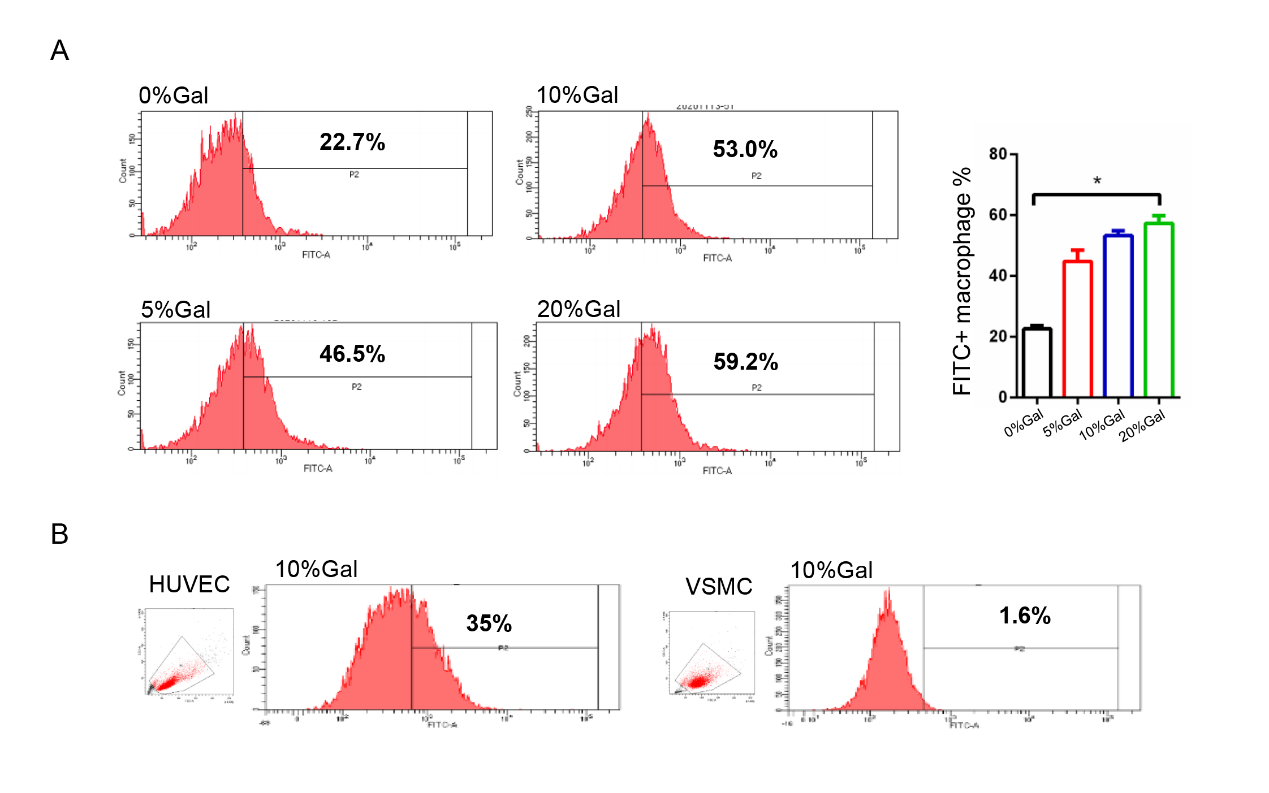
**

**Figure S2. Detection of the phagocytosis efficiency of miR-93-FAM@HSA NPs.** (A) Representative Flow graphs of FITC+ cells indicating the phagocytic efficiency of macrophage for miR-93-FAM@HSA NPs with 0%, 5% 10% and 20% galactose modification. n=3. (B) Phagocytic efficiency of HUVEC or VSMC for miR-93-FAM@HSA NPs with 10% galactose modification. Data were expressed as mean ± SD, *P<0.05, **P<0.01, ***P<0.001.


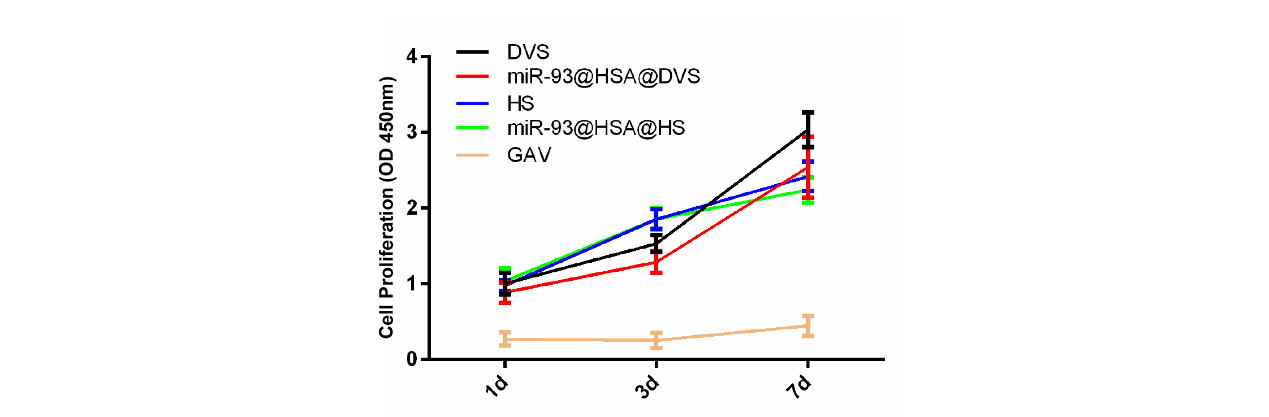


**Figure S3. HUVECs growth curves drawn by CCK-8.** DVS: decellularized valve scaffolds; miR-93@HSA@DVS: miR-93@HSA nanoparticles coated DVS; HS: hydrogel coated DVS; miR-93@HSA@HS: hydrogel and miR-93@HSA nanoparticles coated DVS; GAV: glutaraldehyde crosslinked aortic valve.


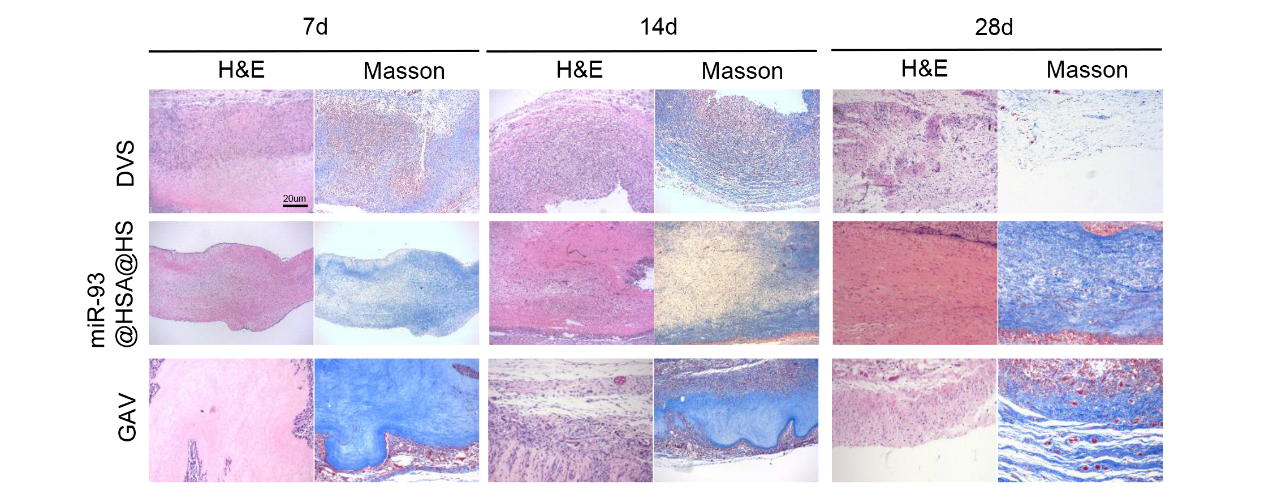


**Figure S4. In vivo histocompatibility after subcutaneous implantation.** representative histological images of the scaffolds after 7-days, 14-days and 28-days implantation. H&E stain indicating cell infiltration. Masson stain indicating collagen degradation. Scale bars = 20 μm. DVS: decellularized valve scaffolds; miR-93@HSA@HS: hydrogel and miR-93@HSA nanoparticles coated DVS; GAV: glutaraldehyde crosslinked aortic valve.


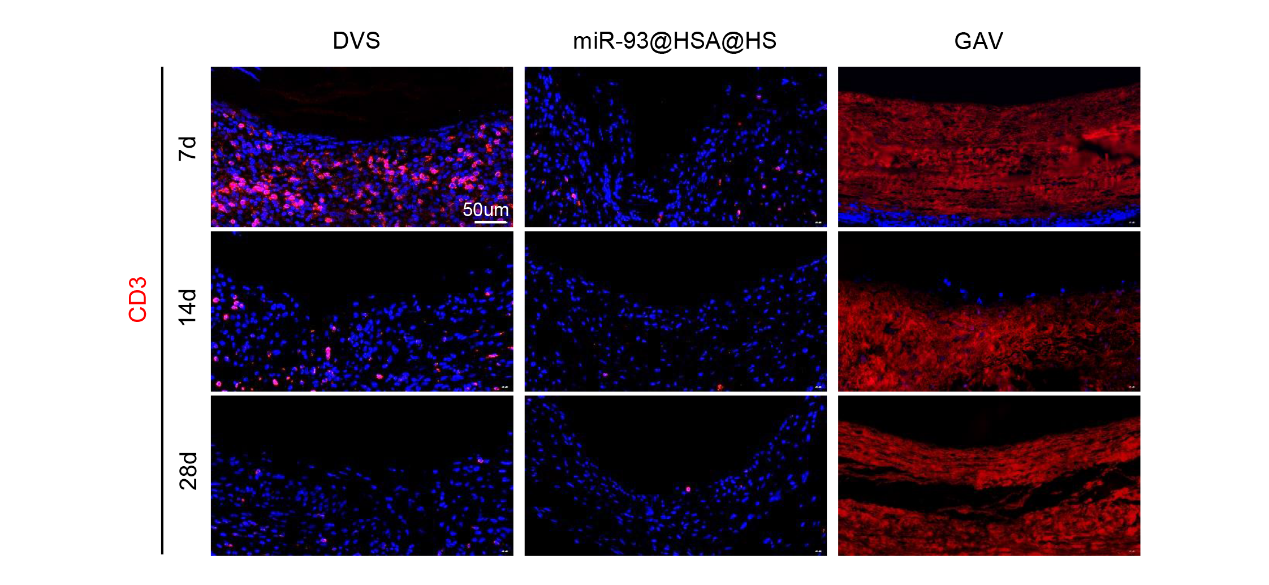


**Figure S5. Immunofluorescence staining of CD3+ T cells in scaffolds after 7-days, 14-days and 28-days transplantation in the rat abdominal aorta.** Scale bars = 50 μm. DVS: decellularized valve scaffolds; miR-93@HSA@HS: hydrogel and miR-93@HSA nanoparticles coated DVS; GAV: glutaraldehyde crosslinked aortic valve.


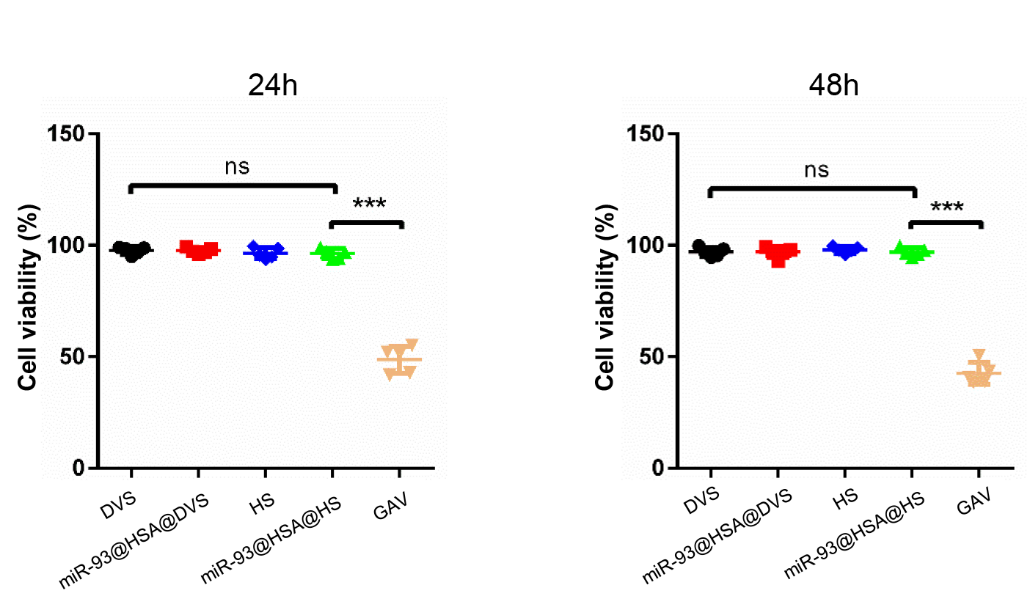


**Figure S6. Cytotoxicity of complex scaffolds.** n=5. Data were expressed as mean ± SD, *P<0.05, **P<0.01, ***P<0.001. DVS: decellularized valve scaffolds; miR-93@HSA@DVS: miR-93@HSA nanoparticles coated DVS; HS: hydrogel coated DVS; miR-93@HSA@HS: hydrogel and miR-93@HSA nanoparticles coated DVS; GAV: glutaraldehyde crosslinked aortic valve.


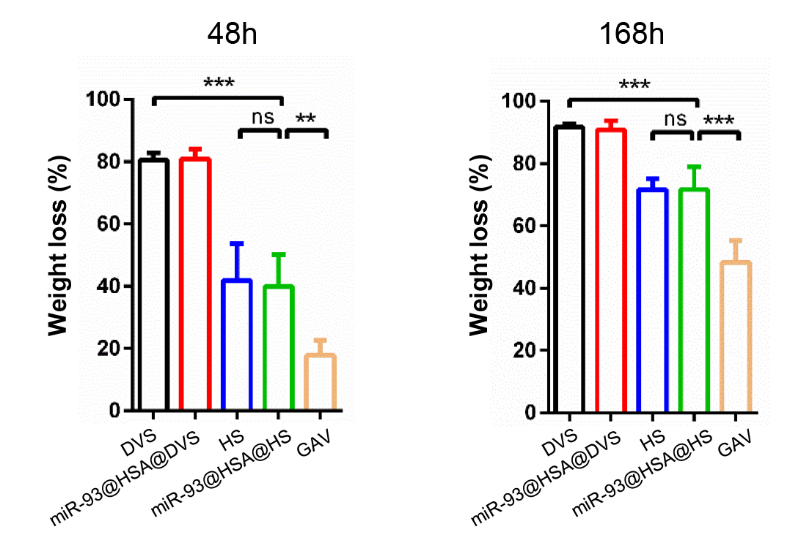


**Figure S7. The relative percentage of weight loss of collagenase solution for 48h and 168h.** n=5. Data were expressed as mean ± SD, *P<0.05, **P<0.01, ***P<0.001. DVS: decellularized valve scaffolds; miR-93@HSA@DVS: miR-93@HSA nanoparticles coated DVS; HS: hydrogel coated DVS; miR-93@HSA@HS: hydrogel and miR-93@HSA nanoparticles coated DVS; GAV: glutaraldehyde crosslinked aortic valve.


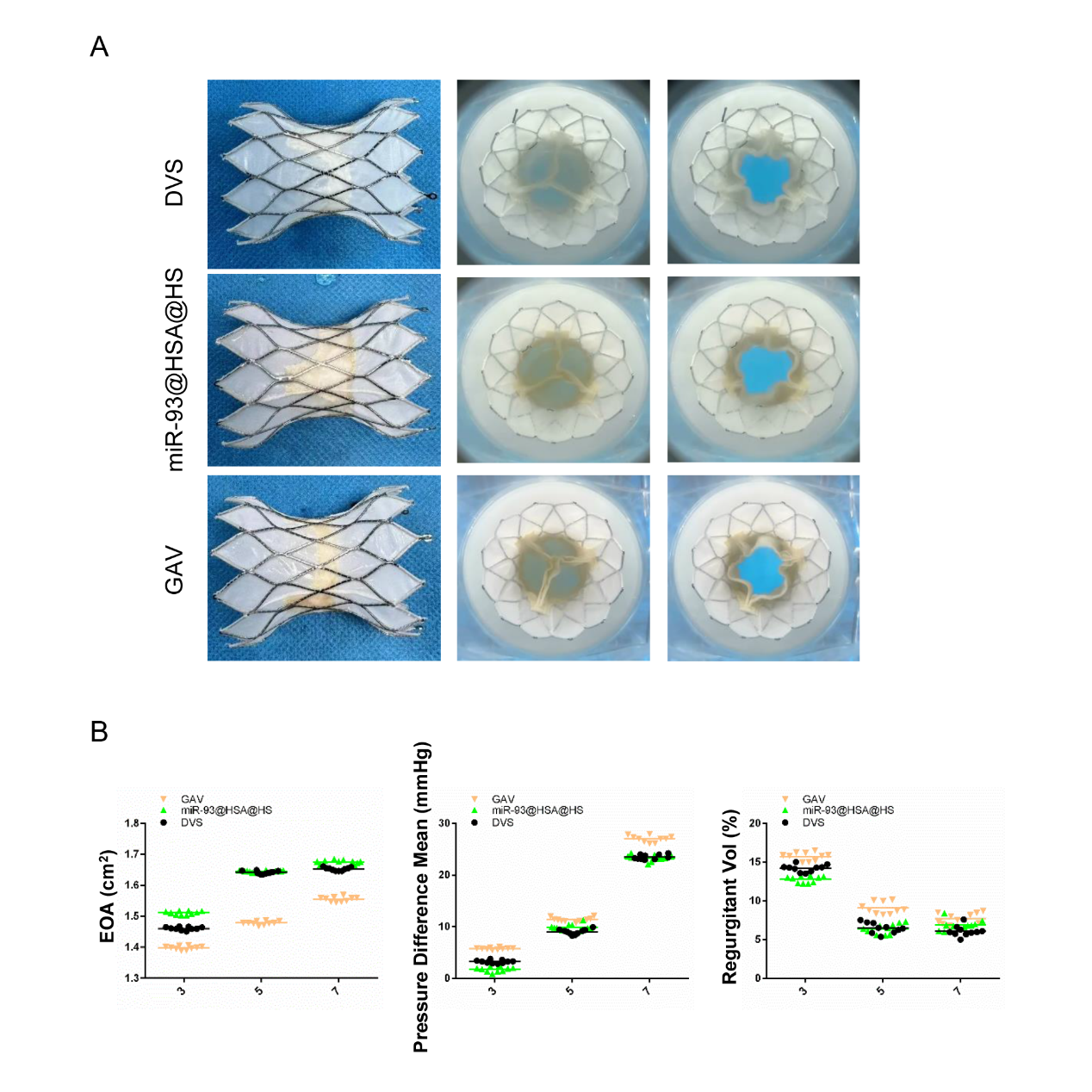


**Figure S8. In vitro pulsatile flow testing.** (A) Representative screenshots showed the state of the valves during the opening and closing cycles. (B) Valve effective orifice area (EOA), regurgitant flow rate, and mean transvalvular pressure difference were measured under conditions of varying cardiac output (3, 5, 7 L/min). DVS: decellularized valve scaffolds; miR-93@HSA@HS: hydrogel and miR-93@HSA nanoparticles coated DVS; GAV: glutaraldehyde crosslinked aortic valve.
